# Literature analysis on asparagus roots and review of its functional characterizations

**DOI:** 10.3389/fnut.2022.1024190

**Published:** 2023-04-17

**Authors:** Yaodong Guo, Zhe Liu, Yingjie Wan, Yanyan Zhang, Hassan Idris Abdu, Meng Yang, Jinjin Pei, Tianli Yue, Xianbin Zhang, Ahmet Hacimuftuoglu, A. M. Abd El-Aty

**Affiliations:** ^1^College of Health Management, Shangluo University, Shangluo, Shaanxi, China; ^2^Shaanxi Key Laboratory of Bioresources, 2011 QinLing-Bashan Mountains Bioresources Comprehensive Development C. I. C., Qinba State Key Laboratory of Biological Resources and Ecological Environment, College of Bioscience and Bioengineering, Shaanxi University of Technology, Hanzhong, Shaanxi, China; ^3^College of Society and Science, Tibet Cultural University, Xianyang, China; ^4^College of Food Science and Technology, Northwest University, Xi’an, Shaanxi, China; ^5^Department of General Surgery, Institute of Precision Diagnosis, Treatment of Digestive System Tumors, Shenzhen University General Hospital, Shenzhen University, Shenzhen, Guangdong, China; ^6^Department of Medical Pharmacology, Faculty of Medicine, Atatürk University, Erzurum, Türkiye; ^7^State Key Laboratory of Biobased Material and Green Papermaking, Qilu University of Technology, Shandong Academy of Sciences, Jinan, China; ^8^Department of Pharmacology, Faculty of Veterinary Medicine, Cairo University, Giza, Egypt

**Keywords:** asparagus roots, physicochemical properties, pharmacology, health benefits, bioactive compounds

## Abstract

Asparagus root (AR) is utilized globally as a traditional herbal medicine because it contains various bioactive compounds, such as polyphenols, flavonoids, saponins, and minerals. The composition profiles of AR are strongly affected by its botanical and geographical origins. Although minerals and heavy metals are minor constituents of AR, they play a crucial role in determining its quality and efficacy. A comprehensive classification of AR, its phytochemistry, and its pharmacology were reviewed and interpreted herein. Potentially eligible articles (in English) were identified through an electronic search of the Web of Science database (2010–2022) and Google (2001–2022). We used the primary search term “Asparagus roots” combined with the words “pharmacology,” “bioactive compounds,” “physicochemical properties,” and “health benefits” to find the relevant literature. We screened the titles, keywords, and abstracts of the publications obtained from the database. A full copy of the article was obtained for further assessment if deemed appropriate. Different asparagus species might potentially be used as herbal medicines and functional foods. Phytochemical studies have revealed the presence of various bioactive compounds as valuable secondary metabolites. The dominant class of bioactive compounds in AR is flavonoids. Furthermore, AR displayed significant pharmacological effects, such as antioxidant, antimicrobial, antiviral, anticancer, anti-inflammatory, and antidiabetic effects, as shown in animal and human studies. This review provides a valuable resource to enable a thorough assessment of the profile of Asparagus root as a functional ingredient for the pharmaceutical and food industries. In addition, it is anticipated that this review will provide information to healthcare professionals seeking alternative sources of critical bioactive compounds.

## 1. Introduction

The Asparagus crop (Family *Asparagaceae*, including approximately 300 species) is native to the eastern Mediterranean region and Asia. The Asparagus crop grows in various soil types (1). Under optimum climate conditions, asparagus could attain growth rates up to 1 cm/h and be harvested once the plant reaches a height close to 21 cm (2). The asparagus plant can be continuously harvested for up to 40 years or until there is a decline in productivity and quality owing to exposure to microbial agents, such as *Fusarium, Phytophthora*, *Stemphylium*, *Phomopsis asparagi (Sacc.) Bubak*, and *Cercospora asparagi Sacc.* species. The regeneration of the rootstock is therefore necessary for sustained productivity. Residual asparagus roots from the main crop are considered waste and are commonly left to rot in fields. However, crops that cannot be degraded after replanting new asparagus plants would cause some problems. The reason is that residual autotoxins present in original asparagus roots might have allopathic properties, which hinder the growth of new asparagus plants (3). This was accounted mainly for *Fusarium*, the primary cause of root rot disease. *Asparagus officinalis*, a versatile plant with a unique flavor, is one of the most popular fresh vegetables worldwide. It was reported that autotoxins in residual asparagus roots might negatively impact sequential crops over time. Hence, physically removing the roots would be better (3). While farmers may be aware of the autotoxicity of asparagus roots (AR), some may consider root removal an unrealistic economic solution due to the costs involved.

However, residual Asparagus roots contain several bioactive compounds, such as saponins, polyphenols, and flavonoids, which have application value in nutraceutical and pharmaceutical processing (4). Most importantly, these bioactive compounds extracted from AR would enable farmers to generate additional revenue from the sale of AR, thus subsidizing the cost of removing the roots (5). On the other hand, removing AR would improve the growth of new asparagus since its allopathic effect was circumvented, thus increasing farmers’ incomes. Such streamlining processes to capture more value from AR *via* the recovery of bioactive compounds with a wide range of health benefits would maximize crop utilization while reducing waste.

Studies on Asparagus and reviews are likely to focus on pharmacological activities because *Asparagus racemosus*, one of the most investigated varieties, has long been used in traditional medicine. Therefore, providing scientific evidence that AR is a valuable byproduct rather than a waste product is crucial to encourage the farming community to ‘harvest’ AR and use it in the nutraceutical and medicinal industries. Therefore, we aimed to provide comprehensive information on the biological activities of major metabolites and the contribution of these compounds in pharmacological and health status to develop AR in the pharmacy and functional food industry. In this review article, potential eligible studies (in English) were identified through an electronic search of the Web of Science database (2010-2022)^1^ and Google (2001–2022). We used the primary search term “Asparagus roots” combined with the words “pharmacology,” “bioactive compounds,” “physicochemical properties,” and “health benefits” to find the relevant literature. We screened the titles, keywords, and abstracts of the publications obtained from the database. A full copy of the article was obtained for further assessment if deemed appropriate.

## 2. Literature analysis of asparagus roots research

CiteSpace software is a visual analysis software developed by Dr. Chenchaomei of Redsell University in the United States, which can be used to analyze the potential multivariate, time-sharing, dynamic and other knowledge in scientific literature. This article takes the Web of Science (WOS) as the core database with ‘Asparagus roots’ as the keyword to search the literature published from 2010 to 2022. The CiteSpace analysis tool and the bibliometric analysis platform^2^ were used to analyze the development status, development trend and research hot spots of asparagus to predict future research trends and provide a reference for the research and development of asparagus.

### 2.1. Analysis of annual documents, research countries and institutes

**FIGURE 1:**
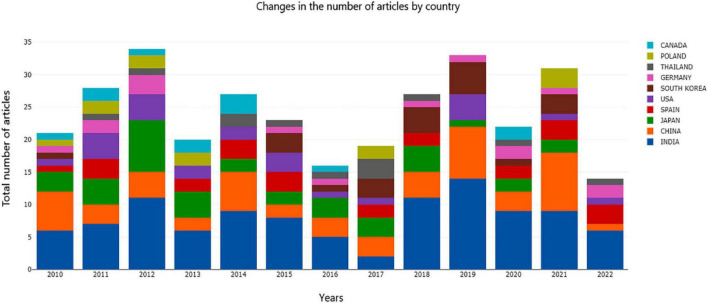
Global document of asparagus roots from 2010 to 2022.

As shown in Figure 1, the top three countries in the number of documents published on AR research were India, China, and Japan in 2010, and in 2021, they were China, India, and Poland. The number of papers published each year ranged from 16 to 34. Research on asparagus has not encountered outbreaks. As the first breeding country and the first exporting country of asparagus, the production of asparagus in China is fairly simple, primarily as a vegetable. Byproduct processing is insufficient, and market efficiency needs to be improved. The roots of asparagus are potential raw materials for functional foods. Therefore, we comprehensively reviewed the research on asparagus roots.

Table 1 was obtained by analyzing the literature of major research institutions in the field of asparagus roots in the world from 2010 to 2022. The research institution with the most papers published is Naresuan University, followed by Gifu University. Interestingly, among the top 10 institutions, none are from China. According to the ranking of total citations, Naresuan University is also the number, followed by Gifu University and Poznań University of Life Sciences. From Table 1, we can see that the published papers on AR were widely distributed to many institutes instead of being deeply researched by several major institutes.

**TABLE 1:** Major research institutions in the field of AR worldwide from 2010 to 2022.

Name of organization	Total number of articles	Total citations	The average number of citations	Total of one play	Number of primes per play	An average is cited
Naresuan Univ	30	61	2.03	6	12	2.00
Gifu Univ	23	31	1.35	12	17	1.42
Poznan Univ Life Sci	19	28	1.47	6	8	1.33
Pusan Natl Univ	16	13	0.81	6	5	0.83
Univ Otago	11	22	2.00	5	14	2.80
CSIC	10	20	2.00	6	11	1.83
Banaras Hindu Univ	10	15	1.50	6	9	1.50
Michigan State Univ	10	12	1.20	8	8	1.00
Univ Malaya	9	8	0.89	2	1	0.50
Hokkaido Univ	8	12	1.50	4	6	1.50

**FIGURE 2:**
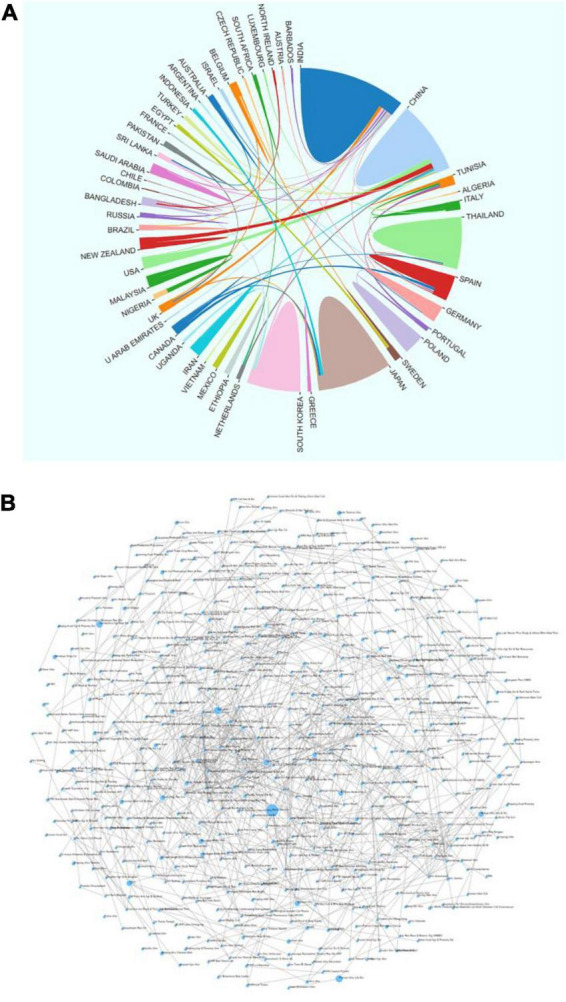
Cooperation between national/regional research institutions of asparagus root (AR) research globally from 2010 to 2022. **(A)** Cooperation between countries and regions; **(B)** cooperation between research institutions.

By analyzing the sending countries and institutions, we can show the distribution, reserves and cooperation of scientific research forces in the field of AR. As shown in Figure 2A, countries cooperate closely in AR; cross-country cooperation mainly focuses on the leading countries, and other countries are the nodes.

India and China are not only at the forefront of AR research but also maintain close cooperation with each other. The cooperation networks with India and China are 11 and 9 countries, respectively. These countries have made outstanding contributions to global AR research.

As seen from the institutional cooperation relationship (Figure 2B), the institutions at the cooperation nodes are the Naresuan Univ, Pozna Univ Life Sci and Univ Otago. From the analysis of research institutions, it can be seen that in the research of AR, one research institution is the center, and other research institutions focus on it. However, the research cooperation links between several major research institutions are slightly insufficient.

### 2.2. Analysis of research direction

**FIGURE 3:**
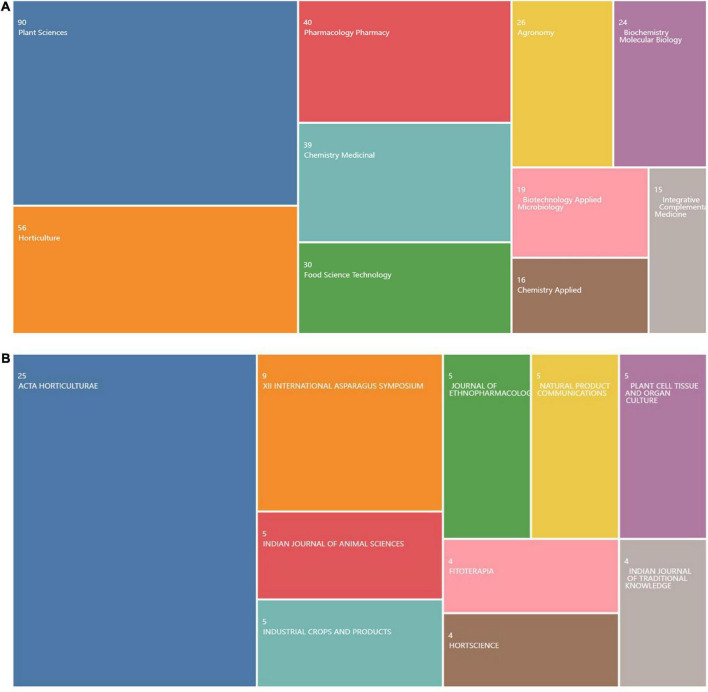
Research direction of asparagus root (AR) from 2010 to 2022. **(A)** Ranking of the research direction of AR in Web of Science (WOS). **(B)** Ranking of the Global Journal publishers of AR.

The research direction in this field can be seen from the amount of literature published in relevant journals. According to the statistical findings of the industry fields of the selected documents in the WOS database (Figure 3). As shown, the research direction is in the three major research fields of Plant Science, Horticulture and Pharmacology Pharmacy (Figure 3B). Through the analysis of the journals published by the selected literature through the online analysis platform of literature metrology, it is found that the top journal is “ACTA Horticulture,” which, with the most papers published and the most frequently cited, is not a top journal. This might suggest that although AR research has been recognized internationally, the academic quality of AR research needs improvement, and the research depth needs to be strengthened.

### 2.3. Analysis of research hotspots

**FIGURE 4:**
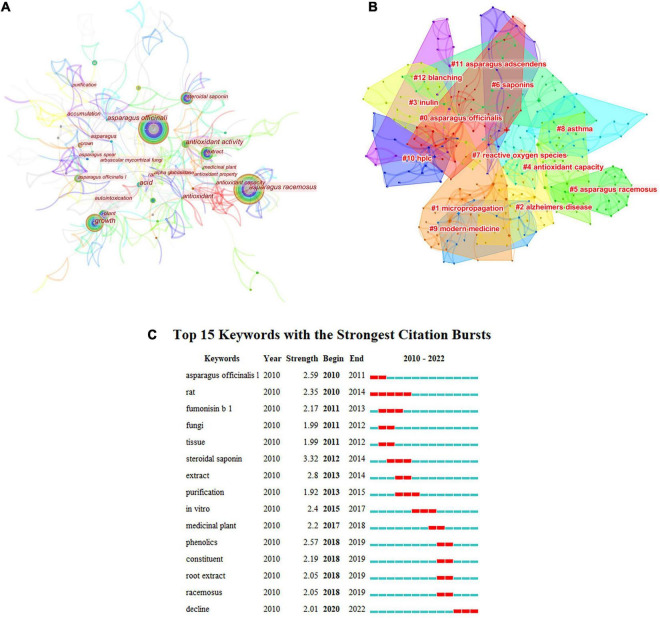
Analysis of keywords. **(A)** Keyword node map of asparagus; **(B)** clustering map of asparagus; **(C)** keyword emergence map.

Research hotspots are the focus that scholars pay attention to and discuss at a certain stage in a specific field. Keywords are the refinement and generalization of the content expressed in an article, so they are often used to analyze the hot spots in a particular field. Figure 4 shows that from 2010 to 2022, the high-frequency keywords for AR research included “antioxidant capacity,” “extract,” “plant growth,” and “antioxidant activity.” This shows that functional research on AR is limited to antioxidants.

Figure 4B shows the cluster analysis of AR keywords in the WOS database. Keyword cluster analysis can further explain the internal relationship between keywords. Through cluster calculation, *q* = 0.8203 > 0.3 and *s* = 0.9328 > 0.7 show effective clustering. Among the 11 clustering modules, the modules related to sturgeon and sturgeon names were removed. AR research has focused on AR bioactivities, such as antioxidants.

Figure 4C shows the emergence analysis of keywords for AR research in the WOS database. From the emergence of keywords, it can be seen that more asparagus plants (*rats*, *fumonisin*, fungi, and so on) were studied in the early stage, and keywords such as root extract and phenolics have gradually appeared since 2018. This also shows that research on asparagus is limited.

## 3. Taxonomy of asparagus

Asparagus is a perennial flowering herb belonging to the genus *Asparagus*, similar to Cousins leek and garlic in the Liliaceae family (2). The Asparagus shoots are classified according to color to white (grown in the absence of light) or green (developed in the presence of light). Generally, asparagus can rise to 100–150 cm and have a stout stem. Asparagus has feathery needle-like leaves. The plant attains a 6–32 mm long and 1 mm wide rosette, clustered of 4–15. Its root type is indeterminate and fascicular. The flower looks bell-shaped, greenish-white to yellowish, 4.5–6.5 mm long, and with six flowers fused at the bottom. They are usually individuals or two or three clusters at the junction of branchlets. They are typically dioecious but sometimes monoecious. The fruits are small red berries (approximately 6–10 mm in diameter) and poisonous to humans. The taxonomy of Asparagus can be summarized as follows:

Kingdom: Plantae
Phylum: Angiospermae
Class: Monocotyledoneae
Tribe: Asparagaceae
Suborder: Lily suborder
Order: Liliaceae
Family: Liliaceae
Genus: Asparagus
Species: *Asparagus officinalis*
Distribution: China, Germany, France, Spain, United States, Japan

## 4. Traditional health benefits of asparagus extract

Many bioactive compounds in asparagus may have health benefits (6, 7). In plants, bioactive compounds may impact numerous functions, such as flavor, color development, and defense systems against ultraviolet (UV) radiation (1). In particular, saponins, polyphenols, and flavonoids have been reported to have health benefits, as summarized in Table 2. For example, it was reported that there are antiglycation (7) and anticancer activities (6) for bioactive compounds from *A. racemosus* roots and antinociceptive and antioxidant effects for *A. officinalis* roots (8).

**TABLE 2:** Summary of health benefits attributed to asparagus extract.

Plant species	Asparagus plant fraction	Health benefit
*A. officinalis*	Roots	Antioxidant effect: The IC50 value for DPPH scavenging activity of the extract (ethanol 70%) (1,117.65 ± 14.26 μg/ml) was significantly higher than that of BHT as control (8) Antinociceptive effects: Formalin and tail-flick test in male Wistar rats exhibited a significant antinociceptive effect at the early stage of the formalin test in the dose of 500 mg/kg intraperitoneally (8).
*A. officinalis*	Roots	Estrogen and progesterone increased in female rats after a 400 mg/kg dose due to increased activity of the hypothalamic-pituitary-gonadal axis (9).
*A. officinalis*	Roots	Anti-diabetic effect: Rats with type 2 diabetes (T2D) showed an improvement after treatment with 500 mg/kg asparagus root extract due to a significant increase in insulin secretion (10).
*A. officinalis*	Spear	Improvement of hypertension (high blood pressure), hyperglycemia (excess glucose in the bloodstream, associated with diabetes), and dyslipidemia (elevates plasma cholesterol) (11).
*A. racemosus*	Roots	Antiglycation: The AGEs inhibitory activity of beta glucogallin isolated from the roots of *A. racemosus* were demonstrated (7). Anticancer: Targeting estrogen receptor α (6).
*A. racemosus*	Shoots	Prevent liver cancer: Rats were given diethylnitrosamine, a carcinogen, and a tumor promoter after a pretreatment with aqueous asparagus roots. The pretreatment prevented hepatocarcinogenesis (12). Galactagogic effect: Deficient lactating women showed significant galactagogic compared to the control. The mechanism of action was attributed to steroidal saponins, which increase prolactin, a protein in mammals that facilitates milk production (13). Antioxidant properties: Good antioxidant when ethanol was used as an extraction solvent. The maximum inhibitory concentration was 78.15 μg/mL (14). Protection against urolithiasis (non-metallic minerals present in the urinary tract): A dose of 800 and 1,600 mg/kg of *A. racemosus* extract significantly reduced serum concentrations of phosphorus, calcium, creatinine, and urea in rats (15).
*A. cochinchinensis*	Roots	Antidepressant Effect: Under stress, the pathway pErK1/2 (extracellular-regulated-kinase) is disrupted, inducing neuronal cell death, in turn, depression. Saponins from asparagus activated the pErK1/2 pathway in rats. This meant that neuron cell function was regulated; hence saponins act as an antidepressant (16). Prevents liver cancer: Saponins in asparagus strongly inhibit liver cancer (HepG2) cells by interacting with reactive oxygen species. This leads to a decrease in the mitochondrial membrane potential, resulting in apoptosis. The IC_50_ was 172.3 μg/mL for HepG2 cells (17). Inhibits HIV-1 Replication: 78% at 20 μg/mL (18). Anti-inflammatory properties: It inhibits inflammation in the ears of mice at 200 mg/kg (19).

Three species of asparagus, including *A. cochinchinensis*, *A. officinalis*, and *A. racemosus*, have been widely studied for their health benefits (6). Rodríguez et al. (20) concluded that most bioactive compounds in asparagus are mainly located in the lower portion of the plant. The majority of published articles on *A. officinalis* (4, 21–23) have predominantly focused on the plant spears (stems) rather than roots as a source of bioactive compounds, as a spear is an edible part and *A. officinalis* is the only species that is commonly eaten. On the other hand, due to its long history of use in folk and traditional medicine, *A. racemosus* has been extensively investigated as a medicinal plant (15).

## 5. Phytochemistry of asparagus root

**FIGURE 5:**
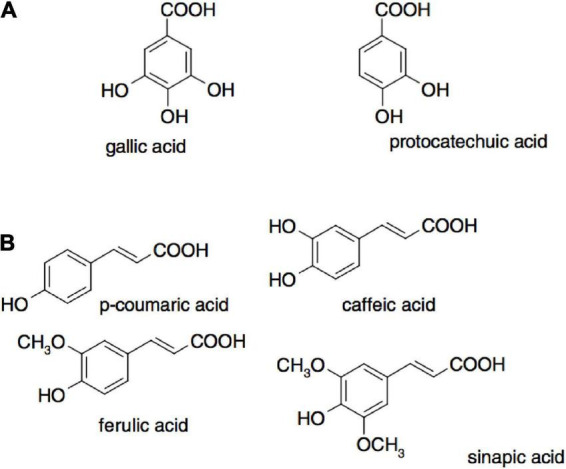
Examples of phenolic acids where **(A)** are hydroxybenzoic acids and **(B)** are hydroxycinnamic acids (38).

Phytochemical compounds, such as ferulic acid, isoferulic acid, malic acid, citric acid, asparagusic acid, caffeic acid, and fumaric acid (Figure 5), are typically extracted from dried *A. officinalis* roots (24, 25). The plant’s origin, species, and portions are important factors when investigating bioactive compounds. Different species have different profiles and quantities of bioactive compounds, and different plant sections contain different concentrations (26). *A. officinalis* roots are a valuable source of fructooligosaccharides (1), which may be isolated and characterized by mass spectrometry (MS), high-performance liquid chromatography (HPLC), NMR, and TLC (24). Furthermore, although common phytochemicals are readily detected *via* classical liquid chromatography, more complex compounds would require gas chromatography–mass spectrometry (GC-MS) for analysis (25). For example, *A. racemosus* roots (ARs) extract subjected to GC-MS analysis showed the presence of 2- furancarboxaldehyde, 1,2-dithiolane-3-carboxylic acid [synonyms: tetranorlipoic acid], 1,6-anhydro-β-d-talopyranase, tetradecanoic acid, n-hexadecanoic acid, oleic acid, 4 H pyran- 4 one, 2,3 dihydro–3,5 dihydroxy–6 methyl, and 9,12- octadecadienoic acid (27). Janani and Singaravadivel (28) confirmed compounds 2-furancarboxaldehyde, 1,2-dithiolane-3-carboxylic acid, 1,6-anhydro-β-d-talopyranase, tetradecanoic acid, n-hexadecanoic acid, oleic acid, 4 H pyran-4 one, 2,3 dihydro-3,5 dihydroxy-6 methyl, and 9,12-octadecadienoic acid in ARs. These results from various studies suggest that the source of AR specifically influences the composition of phytochemical compounds in the roots of various asparagus species and ARs. In another study, the phytochemical compounds found in the ethanol extract of ARs were investigated by Ravishankar et al. (29). The determined compounds were alkaloids, carbohydrates, glycosides, phenolic compounds, tannins, saponins, steroids, and flavonoids. No protein was found in the AR extract. The authors demonstrated that different compounds were extracted in different extraction solvents. For instance, alkaloids, tannins, and proteins were present in the aqueous extracts, whereas steroids and saponins were predominantly found in alcoholic extracts of ARs. Different extraction solvents could solvate different phenolic compounds from ARs, affecting the biological activity of the extracts, e.g., the antibacterial activity (8). Many bioactive compounds (such as steroids, phytosterols, carbohydrates, tannins, anthraquinones, saponins, glycosides, flavonoids, terpenoids, amino acids, and alkaloids) were reported by Ahmad et al. (7) from aqueous extracts of ARs. The results mentioned above suggest that most bioactive compounds in asparagus roots can be dissolved in strongly polar solvents. Furthermore, it appears that the same AR species contain different compositions of bioactive compounds due to regional effects of climate and other environmental factors (30). It is well-documented that biosynthesis of phenolic compounds occurs during photosynthesis, implying that sunshine exposure could affect the composition and amount of individual phenolics. Thus, environmental effects may be a major factor in the bioactive composition in AR. In this context, Dhwaj and Singh analyzed the methanol extracts of ARs and provided a very comprehensive profile of bioactive compounds, e.g., alkaloids, carbohydrates, glycosides, phenolic compounds, tannins, flavonoids, amino acids, proteins, steroids, terpenes, gum, and mucilage (31). Similarly, Sharma et al. isolated and identified a new steroidal sapogenin molecule from ARs and tested its bioactivity in an immune-suppressed animal model that highlighted its potent immunostimulant activity (32). The type of saponins and their composition vary with the asparagus root variety. For instance, Sautour et al. isolated and identified six new steroidal saponins (1–6) from *A. acutifolius L*. roots and evaluated their antifungal activity against human pathogenic yeasts, including *Candida albicans*, *C. glabrata*, and *C. tropicalis*, and noticed a very high activity with a minimum inhibitory concentration (MIC) between 12.5 and 100 μg/ml. Various techniques have been used to purify and identify bioactive compounds in AR (24). Pahwa and Goel. detected 3-heptadecanone, 8-hexadecenoic acid, methyl pentacosanoate, tetratriacontane, tritriacontane, methyl palmitate, tetracosyl tetracosanoate, palmitic acid, stearic acid, asparanin C, asparanin D, asparoside C, asparoside D, 3-β-O-{β-D-2-tetracosylxylopyranosyl}-stigmasterol, 3-β-O-{β-d- glucopyranosyl (1–2)-α-l-arabinopyranosyl}-stigmasterol, β-sitosterol-β-D-glucoside, shatavarin IV, stigmasterol, sarsasapogenin, xanthophyll, ascorbic acid, α-tocopherol, and β-carotene in *A. adscendens Roxb* roots by TLC and high-performance thin-layer chromatography (HPTLC) (33). Sharma et al. (34) identified shatavaroside A/B and 3-0-[α-L-rhamnopyranosyl-(1→2)-α-L-rhamnopyranosyl-(1→4)-0-β-D-glucopyranosyl]-25(S)-5β spirostan-3β-ol as potent immunostimulant compounds from ARs. In another species (35), investigated aspastipuline and 5-hydroxyaspastipuline from *A. stipularis Forssk* roots. Aspastipuline exhibited remarkable cytotoxic activity against the MCF-7 cell line and displayed moderate antiproliferative activity with an IC_50_ (half-maximal inhibitory concentration) value of 4.7 μM. The details mentioned above indicate that a wide range of bioactive compounds with multiple biological functions could be found in AR. This bioactive compound varies in terms of existence and quantity among various ARs due to ecological and genetic factors.

## 6. Bioactive compounds in asparagus

Polyphenols are ubiquitous and are present in plants. They possess various physiological activities, including antiatherogenic, antialgenic, antimicrobial, anti-inflammatory, antithrombotic, and vasodilatory and cardioprotective effects (36). Therefore, there is much interest in plants containing polyphenols, as they can provide several beneficial effects on health attributed to their antioxidant activity (7, 8).

The antioxidant activity of polyphenols is attributed to their ability to donate hydrogen, scavenge free radicals, and chelate metal ions (36). The activity depends on their structure, including the positions and number of hydroxyl groups compared to the carboxyl functional group (37). The main classification of polyphenols includes flavonoids, phenolic acids, tannins, stilbenes, and lignans. The *in vivo* antioxidant activities from asparagus roots are shown in Table 3, and examples of phenolic acids (hydroxybenzoic acids and hydroxycinnamic acids) that exhibit antioxidant activity are presented in Figure 5 (38).

**TABLE 3:** *In vivo* antioxidant activity of asparagus roots.

Sample (Root)	Biological activity	*In vivo* antioxidant activity (Experimental model)					References
		**Animal**	**Method**	**Extraction**	**Dosage**	**Duration**	
*A. racemosus* root	Anti-ulcer	Albino rats (175–225 g)	Total acidity, free acidity, and gastric secretion	Methanol	25–100 mg/kg	5 days	(40)
*A. racemosus* root	Antidiabetic activity	Long-Evans rats (180–250 g)	Glucose, 3-Isobutyl-1-methylxanthine (IBMX), tolbutamide	80% ethanol	40–200 μg/mL		(41, 42)
*A. racemosus root*	Hypolipidemic activity	Male albino rats (Charles Foster, 150–200 g)	High-density lipoproteins (HDL) and decreased plasma total lipid (TL), total cholesterol (TC), low-density lipoproteins (LDL), and atherogenic index (AI)	Dried powder	5–10%	4 weeks	(43)
*A. racemosus root*	Antiurolithiatic activity	Adult male albino Wistar rats	Level of creatinine, calcium, oxalate, and phosphate ions in urine	Ethanol	0.75% ethylene glycol water	28 days	(44)
*A. racemosus root*	Diuretic activity	Inbred albino rats of the Wistar strain	Lipschitz test	Water	800, 1,600, and 3,200 mg/kg	14 days	(45)
*A. racemosus roots*	Neuroprotective Effect	Female Swiss albino mice	Glutathione peroxidase (GPx), GSH assay	Acetone	2–200 mg/kg	2 weeks	(46)
*A. racemosus* root	Anti-tissue activity	Wistar albino mice of either sex, weighing 25–30 g		Methanol	200–400 mg/kg, p.o	24 h	(47)
*A. racemosus root*	Anti-dyspepsia	8 normal healthy male volunteers	Anemia, diabetes, and any abnormality in renal and liver functions	Powder	2 g	3 h	(48)
*A. racemosus root*	Antidiarrheal activity	Inbreed Albino Wistar rats of either sex	Castor oil-induced diarrhea and PGE2-induced enteropooling gastrointestinal motility in charcoal meal test	Ethanol	150, 200, and 250 mg/kg	4 h	(49)
*A. racemosus root*	Anticonvulsant activity	Female Wistar rats (180–200 g)	Hind limb extension, clonus, stupor, the onset of tonic seizures	Chloroform methanol Aqueous	200 mg/kg, p.o 90 mg/kg, p.o	1 h	(50)
*A. cochinchinesis roots*	Anti-inflammatory activity	Specific pathogen-free 5-week-old male	skin thickness and tissue weight, inflammatory cytokine production, neutrophil-mediated myeloperoxidase (MPO) activity, and various histopathological indicators	70% ethanol	200 mg/kg	7 days	(19)

**FIGURE 6:**
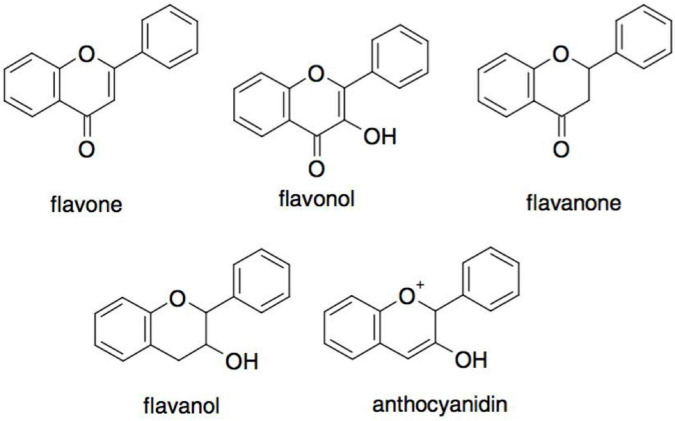
Different major subclasses of flavonoids (38).

The most consumed polyphenols in the human diet are flavonoids. They have a basic structure of a flavan nucleus with 15 carbon atoms arranged in three rings (C6-C3-C6) (39). Depending on the oxidative state of the central C ring, flavonoids can be categorized into five subgroups: flavones, flavonols flavanones, flavanols, and anthocyanins (Figure 6) (38). The antioxidant activity depends on their structure and the availability of hydroxyl groups, which allows them to scavenge radicals. It must be noted that the separation of flavonoids is challenging, as they have similar structures (36).

### 6.1. Polyphenols and flavonoids in asparagus

The phenolic and flavonoid contents in asparagus have been investigated in the literature. Asparagus contains different polyphenols, including hydroxycinnamic and ferulic acids, in moderate amounts as determined by HPLC analysis (51). In this context, Nile and Park (22) found that ARs had a higher polyphenol content of 28.17 GAE/100 g compared to other medicinal plants, such as *Butea monosperma*, *Withania somnifera*, *Tephrosia purpurea*, *Vitex negudo*, and *Plumbago zeylanica*, with polyphenol contents of 24.07 GAE/100 g, 24.02 GAE/100 g, 18.02 GAE/100 g, 12.45 GAE/100 g, and 10.21 GAE/100 g, respectively. This result may indicate that asparagus roots are a good source of polyphenols. Furthermore, some studies have investigated the association between polyphenols and the antioxidant activity of asparagus and found good agreement between the total polyphenols (as determined by HPLC and colorimetric assays) and the antioxidant activity (37).

Solana et al. (52) investigated the flavonoid and phenolic compounds in whole *A. officinalis* and found that rutin is the most abundant phenolic compound. Rutin, also known as sophorin, quercetin-3-rutinoside, and ruthenium, is similar to saponin structures because of its aromatic configuration. Rutin is a glycoside hydrolyzed into aglycone and quercetin by enzymes in the human gut microbiota (53). Rutin has been shown to improve colitis, modulate the signaling of tumor necrosis factor-alpha and nuclear factor kappa B activity, reduce myeloperoxidase activity and modulate the levels of proinflammatory cytokines (54).

Caffeic acid was found to be the main phenolic compound in the methanol extract of fresh *A. officinalis* roots. A wider array of bioactive compounds, including 1,3-0-di-trans-p-coumaroylglycerol (1.7 mg/100 g extract), tetracosanoic acid (12.1 mg/100 g extract), 4′7-dimethylkaempferol (4.9 mg/100 g extract), rutin (211.3 mg/100 g extract), quercetin (7.0 mg/100 g extract), L-asparagine (3.7 mg/100 g extract), caffeic acid (12.0 mg/100 g extract), ferulic acid (5.9 mg/100 g extract), and inosine (5.3 mg/100 g extract), were characterized in *A. officinalis* roots by HPLC and NMR (55).

### 6.2. Lignans, isoflavones, and saponins

AR contains phytonutrients, such as lignans, isoflavones, and saponins, that have health benefits. These phytonutrients may prevent the formation of cancerous tumors, lower blood pressure, reduce the risk of stomach ulcers and slow down the degeneration of cells (39). Phytoestrogens are naturally occurring polycyclic phenols found in AR. There are two classes of phytoestrogens in ARs: isoflavones and lignans (56). Flavonoids may partly contribute to breast cancer prevention due to their antiestrogenic properties. Huang et al. identified rutin and quercetin in *A. officinalis* roots (55). In this study, rutin was obtained for the first time. On the other hand, lignans are dimers of phenylpropanoid (C6-C3) units linked by the central carbons of their side chains. In this context, Huang et al. (56) first separated four lignan types, (+)-nyasol, 3′-methoxynyasin, syringaresinol-4′, 4″-0-bis-β-D-glucoside, and syringaresinol-4-0-β-D-glucopyranoside, from *A. officinalis* roots.

AR also provides opportunities to develop value-added food products that enhance health benefits. Li et al. (57) found that the total saponin content in *A. officinalis* roots was 3.6 times higher than that in *A. officinalis* spears. Moreover, Shatavarin saponin glycosides from ARs have shown moderate protection against Cu^+^-induced oxidation of human low-density lipoprotein (LDL) with no inhibition of lipid peroxidation as determined by thiobarbituric acid reactive assay (TBARs) (58). Isoflavones, asparagamine, racemosol, polysaccharides, and mucilage were also reported by Chawla et al. (59).

**FIGURE 7:**
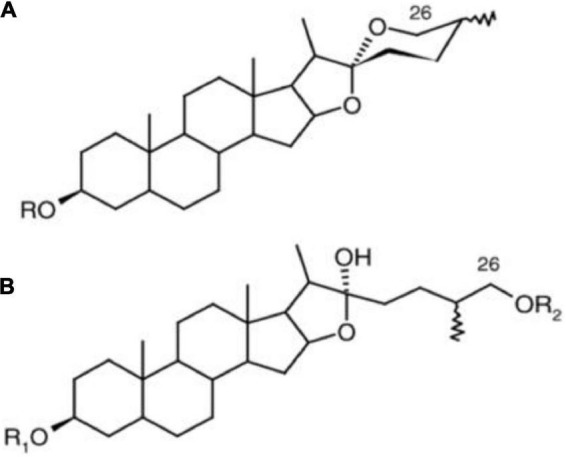
Different structures of steroidal saponins **(A)** steroidal spirostane and **(B)** steroidal furostane, where R = sugar moiety (62).

Saponins naturally occur as glycosides in plant tubers, roots, leaves, and seeds. Over 100 plant families contain saponins, and studies have shown that at least 150 types of saponins have significant anticarcinogenic properties (60). Saponins are characterized by soap-like foaming properties and their sweet or bitter taste (depending on the saponin type) (61). Depending on their chemical structure, saponins can be grouped under many classifications: tirucallanes, dammaranes, hopanes, lupanes, taraxasternaes, oleananes, cycloartanes ursanes, lanostanes, steroidal compounds, and cucurbitanes (Figure 7) (62). These different classifications of saponins are due to other positions, types, and amounts of sugar moieties attached *via* a glycosidic link. Hence, the hydrophobic and hydrophilic components of the compound are responsible for the ability of saponins to have soap-like properties.

#### 6.2.1. Saponins health properties

Saponins can act as anti-carcinogens as they force cancerous cells to undergo apoptosis (60). Apoptosis is a natural form of cell death, as it instructs the cell to self-destruct. This is different from necrosis, which is caused by external factors and has adverse side effects. Therefore, saponins have been seen as ideal candidate drugs to treat cancer. Saponins appear to prevent a wide range of cancers, as highlighted in Table 2. The literature has described saponins as vital in folk medicine (61). They provide antioxidant, anti-osteogenic, and anti-inflammatory effects.

#### 6.2.2. Saponins in asparagus

**FIGURE 8:**
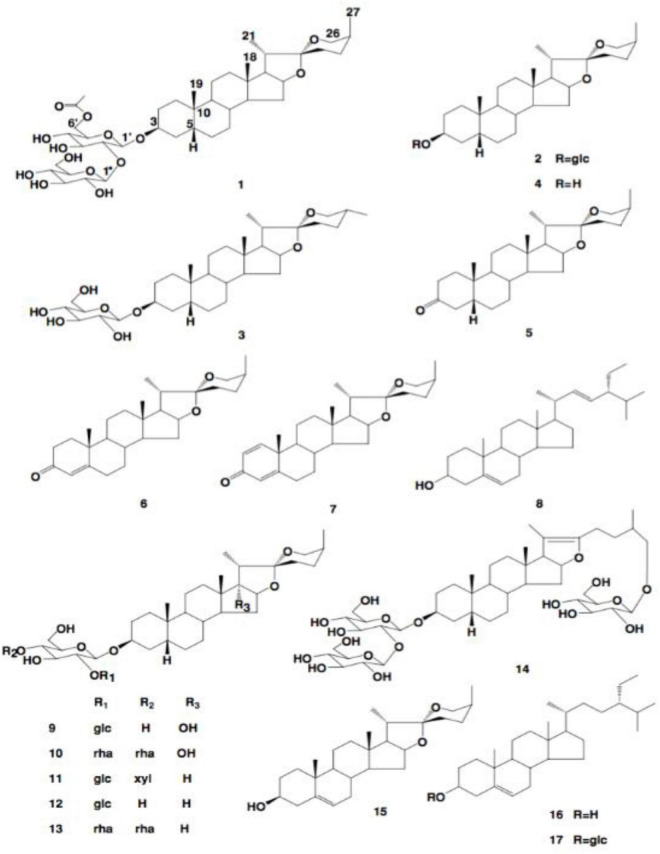
The structures of saponins **1–18** (63).

In asparagus, the main type of saponin present is steroidal (60). Steroidal saponins contain a steroidal aglycone with a 27-carbon chain skeleton in a six-ring structure. In some plant sources, in the 26th position, the hydroxyl group is occupied in a glycosidic bond, maintaining the aglycone structure as pentacyclic (63). These structures are shown in Figure 8.

There is a vast amount of research on the health benefits of saponins extracted from asparagus. A potential reason for the wide range of health benefits could be the ability of saponins to interact with reactive oxygen species (64). As saponins exhibit aglycone moieties, many different saponins are present in the asparagus root and spear (60). Huang et al. found seventeen other saponins in *A. officinalis* roots (Table 4) (55). They also determined the structures of these saponins. These structures are presented in Figure 8. Saponins **1–17** have an aromatic configuration with different side chains depending on the type of compound. Huang et al. also investigated the *in vitro* cytotoxicity of these saponins against various cancer cells (65). Specifically, saponin **1** had an IC_50_ value of 5.50 μM against a nasopharyngeal CNE cancer cell line; saponin **11** had an IC_50_ value of 1.46 μM against a mouse leukemia L1210 cell line; and saponin **12** was effective against a wide range of cancer cells, including esophageal Eca-109, leukemia L1210, gastric MGC-803, nasopharynx KB, and lung LTEP-a-2 cells, with IC_50_ values of 4.03, 4.32, 3.72, 1.38, and 3.16 μM, respectively. Therefore, saponins **1**, **11,** and **12** had significant inhibitory effects against tumor cell proliferation, and saponins **2**, **3**, **4**, **6**, and **13** inhibited tumor cells moderately (55). Lee et al. (66) investigated the cytotoxic effects of *A. cochinchinensis* root extracts and found a new saponin, methyl protodioscin (**18**), responsible for inhibiting A549 lung cells with an IC_50_ of 59.1 μM. This saponin was also found to reduce cholesterol levels in rats.

**TABLE 4:** Isolated phytochemicals from the roots of asparagus species.

Sample	Isolated compounds	Content	Methods of analysis and identification	References
*A. Officinalis L*	Sarsasapogenin M	16 mg C_39_H_64_O_14_	Cytotoxic activities against human and mouse tumor cell	(55, 56)
	Sarsasapogenin N	10 mg C_45_H_74_O_17_		
	(25S)-5β-spirostan-3β-ol-3-O-β-D-glucopyranosyl-(1,2)-[β-D-xylopyranosyl-(1,4)]-β-D-glucopyranoside	42 mg	A2780, HO-8910, Eca-109, MGC-803, CNE, LTEP-a-2, KB and mouse L1210 tumor cells.	
	(25S)-5β-spirostan-3β-ol-3-O-β-D-glucopyranosyl-(1,2)-β-D-glucopyranoside	63 mg	Gel column chromatography, NMR, RP-HPLC, LC-MS,	
	(25S)-5β-spirostan-3β-ol-3-O-α-L-rhamnopyranosyl-(1,2)-[α-L-rhamnopyranosyl-(1,4)]-β-D-glucopyranoside	23 mg C_45_H_74_O_16_		
	(25S)26-O-β-D-glucopyranosyl-5β-furost-20 (22)-ene-3β,26-diol-3-O-β-D-glucopyranosyl-(1,2)-β-D-glucopyranoside	650 mg		
	Yamogenin	47 mg		
	β-sitosterol	1.2 mg		
	Sitosterol-β-D-glucoside	2.3 g		
	D-glucose	–		
	Asparagoside A	–		
	(25*R*)-5β-spirostan-3β-ol 3-*O*-β-D-glucopyranoside (3)	–		
	Sarsasapogenin (4)	–		
	Sarsasapogenone (5)	–		
	(25*S*)-neospirost-4-en-3-one (6)	–		
	25*S*-spirosta-1,4-dien-3-one (7)	–		
	Stigmasterol (8) A -D-fructofuranose-1,2′:2,1′-β-D-fructofuranose dianhydride	22 mg C_12_H_20_O_10_		
*A. Officinalis L*	1,3-O-di-trans-p-coumaroylglycerol	12 mg C_21_H_20_O_7_		(55, 56)
	Tetracosanoic acid	86 mg		
	4′7-dimethylkaempferol	35 mg		
	Rutin	1,500 mg		
	Quercetin	50 mg		
	5-hydrox-ymethyl-furaldehyde	18 mg		
	L-asparagine	26 mg		
	Caffeic acid	85 mg		
	Ferulic acid	42 mg		
	Inosine	10 mg		
	n-butyl-β-D-fructofuranoside	430 mg		
	Ethyl-β-D-fructopyranoside	25 mg		
	Sucrose	16 mg		
*A. Officinalis L*	(+)-nyasol	56 mg		
	3′-methoxynyasin	27 mg		
	Syringaresinol-4′,4″-O-bis-β-D-glucoside	185 mg		
	Syringaresinol-4-O-β-D-glucopyranoside	21 mg		
*A. Officinalis L*	1^F^-β-D-fructofuranosyl-4^G^-β-D-galactopyranosylsucrose, *O*-β-D-galactopyranosylsucrose, -fructofurano-syl-*O*-[ructofurano-syl-sylsucros)]-α-D-glucopyranoside	Saccharide A 120 mg 9.5% yield from the donor saccharide	*Clostridium perfringens*, *Escherichia coli*, and *Enterococcus faecalis* Gas-liquid chromatography, paper, and thin-layer chromatography, anion-exchange chromatography Gel permeation chromatography (GPC), DEAE-, CM-cellulose, Sephadex G-200, and sucrose-coupled sepharose 6B.	(67)
	1^F^(1-β-D-fructofuranosyl)_2_-4^G^-β-D-galactopyranosylsucrose, [*O*-β-D-galactopyranosylsucros_2_-β-D-fructofuranosyl-*O*-[-D-fructofuranosyl-sucrose, [from the donor sac	Saccharide B 19 mg 1.5% yield from the donor saccharide		
	1^F^ (1-β-fructofuranosyl)_1_-kestose sucrose	0.5 g		
	1^F^ (1-β-fructofuranosyl)_2_-nystose sucrose	1.13 g		
	1^F^ (1-β-fructofuranosyl)_3_	0.25 g		
	6^G^(1-β-fructofuranosyl)1-neokestose sucrose	0.20 g		
	6^G^(1-β-fructofuranosyl)_2_ sucrose	0.93 g		
	6^G^(1-β-fructofuranosyl)_3_ sucrose	0.66 g		
	1^F^,6^G^-di-β-fructofuranosyl sucrose	1.10 g		
	1^F^(1-β-fructofuranosyl)_2_-6^G^-β-fructofuranosyl sucrose	7.06 g		
*A. cochinchinensis*	Asparacoside	771 mg		(68)
	Asparacosins A	372 mg		
	Asparacosin B	18.2 mg		
	3″-methoxyasparenydiol	9.6 mg		
	asparenydiol	2.0 mg		
	3′-hydroxy-4′-methoxy-4′-dehydroxynyasol	2.6 mg		
	Nyasol	5.6 mg		
	3″-methoxynyasol	5.7 mg		
	1,3-bis-di-p-hydroxyphenyl1-4-penten-1-one	2.3 mg		
	*trans*-coniferyl alcohol	3.5 mg		
*A. gobicus*	Norlignans	–	Cytotoxic	(69)
*A. meioclados*	(Asparosides A) 23-O-α-arabinopyranosyl-(5β, 25S)-spirostan-3β, 23α-diol-3-O-{β-D-xylopyranosyl(1→4)}-β-D-glucopyranoside, and (Asparosides B) 26-O-β-glucopyranosyl-5β-furost-20(22)-ene-3β,	–	TLC, NMR	(70)
	26-diol-3-O-{β-D-xylopyranosyl(123-O-α- arabinopyrano			
*A. acutifolius*	(25S)-3β,5β,22α-22-methoxyfurostane-3,26-diol 3-*O*-β-D- xylopyranosyl-(122-m[β-D- xylopyranosyl-(1→4)- β-D-glucopyranosyl 26-*O*-β-D-glucopyranoside	C_50_H_84_O_22_ 9 mg	Antifungal activity	(71)
	(25S)-3β,5β,22α-furostane-3,22,26-triol 3-*O*–5S)-3β,5β,22α-furostane [β-D-xylopyranosyl -(1→4)- β-D-glucopyranosyl 26-*O*-β-D-glucopyranoside	C_49_H_82_O_22_ 6 mg	NMR, TLC	
	(25S)-3β,5β,22α-22-methoxyfurostane -3,26-diol 3-*O*-25S)-3β,5β,22α-22-methox [β-D-glucopyranosyl 26-*O*-β-D-glucopyranoside	C_45_H_76_O_18_ 6 mg	*C. albicans*, *C. glabrata* and *C. tropicalis*	
	(25S)-5β-spirostane-3β, 17α-diol 3-*O*-25S)-5β-spirostane-3β, 1 [β-D- xylopyranosyl-(1→4)]-β-D-glucopyranoside	C_43_H_70_O_17_ 8 mg		
	(25S)-5β-spirostane-3β-ol 3-*O*-25S)-5β-spirostane-3β-ol [β-D-xylopyranosyl -(1→4)]-β-D-glucopyranoside	C_43_H_70_O_16_ 8 mg		
	(25S)-5β-spirostane-3β, 17α-diol 3-*O*-β-D-glucopyranosyl-(1→2)- [β-D- xylopyranosyl-(1→4)]-β-D-glucopyranoside	C_44_H_72_O_18_ 7 mg		
*A. racemosus*	Shatavarin I	8.2 mg	RP-HPLC, 1D,2D NMR, LC-MS	(72, 73)
	Shatavarin VI	3.2 mg		
	Shatavarin VII	0.5 mg		
	Shatavarin VIII	1.6 mg		
	Shatavarin IX	1.1 mg		
	Shatavarin X	0.3 mg		
	ShatavarinV	19.4 mg		
	Asparanin A	0.61 mg		
	Shatavarin V	1.3 mg		
*A. racemosus*	Immunoside	1.8 mg	Anti-lipid peroxidation ELISA, and LC–MS/MS	(58)
	Asparacoside	–		
	Shatavarin IX	–		
	Asparanin A			
	Glucose-arabinose derivative of (25s)-5β-spirostan-3βyl-D-glucoside	–		
	Shatavarin V	–		
	Arabinose-arabinose derivative of (25S)-5β-spirostan-3β-D-glucoside	–		
	arabinose derivative of (25S)-5β-spirostan-3β-yl β-D-glucoside	–		
	Shatavarin X	–		
*A. racemosus*	Alkaloids	–	DPPH further isolated flavonoids, characterized by chemical tests, TLC, and spectral studies, such as UV, FT-IR, 1H-NMR, and LC-MS.	(74)
	Steroids			
	Carbohydrates			
	Flavonoids			
	Tannins			
	Saponins		Toxicity study	
	Proteins and amino acids			
*A. racemosus*	Asparagamine A	3.56 g	Immunomodulator	(75)
	Racemosol	152 mg		
	Racemofuran	146 mg		
	3-0-[cemosus Aopyranosyl-(1A2)-ranosyl-(1Ayranosyl-(1(1A-0-nosyl-(1(1Aranosyl]-25(S)-5a spirostan-3β-ol	–		
*A. racemosus*	(1S,2R,3S,8S,9S,10S,13S,14S,16S,17R,22R,25R)-21-nor-18β, 27α-dimethyl-1β, 2β, 3β-trihydroxy-25-spirost-4-en-19β-oic acid. 1	–	NMR A potent immunostimulant	(32)
*A. racemosus*	Steroidal M	15 μ g/mL	Anti-HIV activity	(76)
	H	NS		
	C	20		
	E	15		
	B	40		
	Ag	40		
*A. racemosus*	Shatavaroside A	30 mg	Potent immunostimulant NMR, HP-20	(34)
	Shatavaroside B	42 mg		
*A. racemosus*	Isoflavone, 8-methoxy-5,6,4′-trihydroxyisoflavone-7-O-β-D-glucopyranoside	0.082%	NMR	(77)
*A. racemosus*	3-O-{[emosusucopyranosyl(1-2)] [ucosidemnopy- ranosyl(1-2)][ucosidecopyranosyl}-26-O-(ucosid-ecopyranosyl)-(25S)5ucosidetan-3anosyl)6-triol,	19% of the total saponin	RP-HPLC, NMR	(78)
*A. racemosus*	Asparagamine A	DPPH	Identification of antioxidant activity	(79)
	Racemosol			
	Racemofuran			
*A. adscendens Roxb*	3-heptadecanone	–		(33)
	8-hexadecenoic acid	–	Thin-layer chromatography (TLC)	
	Methyl pentacosanoate	–	HPTLC	
	Tetratriacontane	–		
	Tritriacontane	–		
	Methyl palmitate	–	Antitoxicity activity	
	Tetracosyl tetracosanoate	–	Antioxidant	
	Palmitic acid	–		
	Stearic acid	–		
	Asparanin C	–		
	Asparanin D	–		
	Asparoside C	–		
	Asparoside D	–		
	3-β-O-{β-D-2-tetracosylxylopyranosyl}-stigmasterol, 3-β-O-{β-d-glucopyranosyl (1–2)-α-l-arabinopyranosyl}-stigmasterol,	–		
*A. stipularis Forssk*	Aspastipuline		The cytotoxic activity of aspastipuline against the MCF-7 cell line NMR.	(35)
	5-hydroxyaspastipuline			
*A. filicinus*	Furostanoside (aspafilioside D), officinalisnin II, and tormentic acid	–	NMR, D-101 macroporous resin, MCL gel CHP-20P, and Sephadex LH-20	(80)

### 6.3. Minerals value in asparagus root

Trace elements such as Ca, Mg, Fe, copper (Cu), and zinc (Zn) are present in AR (81). The trace elements from AR are summarized in Table 5. Several studies have investigated the trace elements in AR cultivars, including *A. officinalis*, *A. racemosus*, and *A. curillus* roots, such as Mg, P, potassium (K), and sodium (Na) consumed at levels of >100 mg/day for a healthy body (Table 6). In contrast, microminerals such as Cu, Fe, Zn, cobalt (Co), and manganese (Mn) are only needed in minimal quantities (<100 mg/day) (82). It was found that the concentration of trace elements was influenced by different altitudes and parts of the entire plant (83). Negi et al. reported that *A. curillus* (Buch.-Ham.) ex-Roxb roots exhibited the highest concentrations of Zn, Cu, Na, K, Ca, and Li, but Mn, Fe, and Co were the highest in *A. curillus* leaves (84). Meanwhile, the concentrations of Cu and Mn were low, Zn, Co, Na, and lithium (Li) concentrations were moderate, and K, Ca, and Fe concentrations were very high in *A. curillus* roots collected from four different altitudes in three seasons. Therefore, the results show that AR is a promising source of extracts that can treat many diseases (85). In another study, Li et al. reported that Ca in *A. officinalis* roots was 7.5 times higher than that in *A. officinalis* spears. In comparison, the content of Fe in *A. officinalis* roots was 15 times higher than that in *A. officinalis* spears (86). The presence of Ca in the AR powder correlates with the analytical results for galactagogues.

**TABLE 5:** Cytotoxicity of saponins and minerals obtained from *A. officinalis* and *A. cochinchinensis* roots.

Compound	IC_50_ values (μM)								
	Ovarian		Esophageal ECA-109^20^	Gastric MGC-803^20^	Nasopharyngeal CNE^20^	Lung		Nasopharynx KB^20^	Leukemia L1210^20^
	A2780^20^	HO-8910^20^				LTEP-a-2^20^	A549^21^		
Sarsasapogenin O (65)	10.57	–	–	8.39	5.50	–	–	–	–
Asparagoside A (65)	10.22	5.04	–	–	–	–	–	–	–
(25*R*)-5β-spirostan-3β-ol 3-*O*-β*-*D-glucopyranoside (65)	–	24.83	–	–	–	–	–	–	12.33
Sarsasapogenin (65)	6.09	–	–	–	–	–	–	–	–
Sarsasapogenone (65)	–								
(25*S*)-neospirost-4-en-3-one (65)	18.85	–	–	–	–	–	–	–	–
25*S*- spirosta-1,4-dien-3-one (65)	–								
Stigmasterol (65)	–								
Sarsasapogenin M (65)	–								
Sarsasapogenin N (65)	–								
(25*S*)- 5β-spirostan-3β-ol 3-*O-*β*-*D-glucopyranosyl-(1→2)-[β*-*D-xylopyranosyl-(1→4)]-β-D-glucopyranoside (65)	–	–	2.91	–	–	2.85	–	2.66	1.46
(25*S*)-5β-spirostan-3β-ol 3-*O-*β*-*D-glucopyranosyl-(1→2)-β-D- glucopyranoside (65)	–	–	4.03	3.72	–	3.16	–	1.38	4.32
(25*S*)-5β-spirostan-3β-ol 3-*O-*α*-*L*-*rhamnopyranosyl-(1→2)-[α-L*-*rhamnopyranosyl-(1→4)]-β-D-glucopyranoside (65)	–	–	10.15	–	12.88	–	–	–	–
(25*S*)-26-*O-*β*-*D-glucopyranosyl-5β- furost-20(22)-ene-3β,26-diol 3-*O-*β-D-glucopyranosyl-(1→2)-β*-*Dglucopyr-anoside (65)	–								
Vamogenin (65)	–								
β-sitosterol (65)	–								
Sitosterol-β-D-glucoside (65)]	–								

**TABLE 6:** Contents of trace elements in AR.

Name of AR	Trace elements	Units	Contents	References
	Co	mg/kg	91.0	
	Na	mg/kg	381.0	
	K	mg/kg	9,508.0	
	Ca	mg/kg	3,076.0	
	Li	mg/kg	78.0	
*A. racemosus* root	Zn	mg/kg	165.0	(84)
	Cu	mg/kg	34.0	
	Mn	mg/kg	84.0	
	Fe	mg/kg	2,040.0	
	Co	mg/kg	122.0	
	Na	mg/kg	745.0	
	K	mg/kg	13,260.0	
	Ca	mg/kg	6,153.0	
	Li	mg/kg	58.0	
*A. officinalis* root	Ca	–	–	(86)
	Fe	–	–	
*A. africanus* root	Zn	–	–	(88)

## 7. Pharmacological activities

### 7.1. Protection against stomach ulcers

Stomach ulcers, commonly known as peptic ulcers (including gastric and duodenal ulcers), are common gastrointestinal disorders affecting millions of people. It is rarely fatal (89). Many herbal drugs are used as ayurvedic preparations and in Indian traditional medicinal systems to manage peptic ulcers (90). The methanolic extract of *A. racemosus* root indicated antisecretory. Antiulcer activity against a non-steroidal anti-inflammatory drug (indomethacin) and pyloric ligation (PL) that induced gastric ulcers in rats showed that crude extracts significantly reduced total acidity, free acidity, and gastric secretion at doses of 25–100 mg/kg for 5 days and protected rats considerably against duodenal ulcers at a dose of 50 mg/kg, twice daily (91). This suggests that *A. racemosus* roots have enhanced mucosal defensive factors, including the life span of cells, mucus secretion and cellular mucus, and antioxidant effects.

### 7.2. Insulin secretory activity (antidiabetic activity)

Herbal medicines are cheaper than modern synthetic drugs for treating widespread and rapidly growing diseases. The evaluation of more herbal plants for the treatment of diabetes is increasing. A study by Hannan et al. (92) reported that the ethanol extract of *A. racemosus* root significantly improved insulin secretion from the perfused pancreas. Therefore, *A. racemosus* root might be used to treat diabetes in clinical settings (93).

### 7.3. Hypolipidemic activity

Coronary heart diseases are caused by plasma cholesterol, high low-density lipoprotein-cholesterol, and triglyceride levels in the blood, with hyperlipidemic/hypercholesterolemia constituting the major risk factor for atherosclerosis and cardiovascular diseases (94). Cholesterol, along with the generation of reactive oxygen species (ROS), raises serum lipid levels and plays a vital role in the causation of coronary artery disease (CAD) and atherosclerosis. The roots of *asparagus* have been shown to reduce cholesterol levels (95). To investigate the antihypercholesterolemia activity of *A. racemosus* roots, a previous study induced hypercholesterolemia in 3-month-old male albino rats with an atherogenic diet, including 0.75 g% cholesterol and 1.5 g% bile salt in a regular diet (43). It was concluded that hyperlipidemia treated at 5 and 10 g% doses of AR powder for 4 weeks led to significantly increased plasma high-density lipoprotein (HDL) fractions (*P* < 0.05, 19–27%) and decreased total plasma lipid (TL) (14–21%), total cholesterol (TC) (25–35%), low-density lipoproteins (LDL) (32–45%) and AI (atherogenic index) (37–49%) compared to the standard drug atorvastatin. In addition, AR root enhanced the concentrations of SOD, hepatic catalase, and ascorbic acid in hypercholesteremic rats, which might be due to the presence of bioactive compounds, including steroids, flavonoids, glycosides, alkaloids, and phenolic compounds, as confirmed by HPTLC (59).

### 7.4. Antiurolithiatic activity

Ethylene glycol is formed at high concentrations of these ions (calcium, phosphate, and oxalate), contributing to renal stone formation. For example, the ethanolic extract of *A. racemosus* root significantly reduced the elevated creatinine levels, calcium, oxalate, and phosphate ions in the urine of adult male albino Wistar rats fed 0.75% ethylene glycol water. It also increased the urinary level of magnesium, a crystallization inhibitor. These results were supported by histopathological findings that presented signs of improvement after treatment with the ethanolic extract (44).

### 7.5. Effect on diuretic activity

ARs treat acute toxicity and diuretic activity (45). A decoction of AR treated urinary tract infections in ancient times (96). Evaluation of acute toxicity and diuretic activity was carried out by feeding Wistar albino rats 800, 1,600, and 3,200 mg/kg AR water extract (45). The Lipschitz test evaluated the diuretic activity compared with the standard drug furosemide. Urine was collected by housing each animal in separate metabolic cage output at 24 h and was analyzed by colorimetry. The urine electrolytes increased significantly in P^+^, K^+^, and Cl^–^ concentrations at 3,200 mg/kg body weight, higher than the standard drug furosemide. It was also evaluated that animals showed no behavioral, autonomic, or central nervous system changes even at the maximum single dose of 3,200 mg/kg. Phytochemical screening of ARs revealed steroidal saponins, shatavarins I, II, III, and IV, and isoflavones (72).

### 7.6. Antitussive activity

In another study, *A. racemosus* roots were tested for antitussive activity. Jaiswal et al. (97) prepared a methanol extract of *A. racemosus* roots at 200 and 400 mg/kg doses, which was used to recover sulfur-induced coughing in Wistar albino mice. The authors also produced 40 and 58.5% inhibition of sulfur-induced cough at doses of 200 and 400 mg/kg compared with the inhibition of codeine phosphate of 36 and 55.45% at 10 and 20 mg/kg doses. Therefore, *A. racemosus* roots showed significant antitussive activity at 200 and 400 mg/kg (97).

### 7.7. Anti-dyspepsia and antidiarrheal activity

*Asparagus racemosus* is also used in Ayurveda for treating dyspepsia because it possesses an effect comparable to the allopathic drug metoclopramide. Metoclopramide is used to decrease gastric emptying time in patients diagnosed with dyspepsia (48). Diarrhea occurs widely in developing countries and is a fatal disease, killing approximately 2.2 million people per year worldwide, most of whom are infants and children below the age of 5 years (49). Therefore, ethanolic and aqueous extracts of ARs were investigated for antidiarrheal activity against castor oil-induced diarrhea in rats compared to Loperamide (a traditional medicine for treating diarrhea as a positive control) (49). The release of ricinoleic acid from castor oil results in inflammation and irritation of the intestinal mucosa, causing prostaglandins, which stimulate motility and secretion. It is well-known that prostaglandin E causes diarrhea in experimental animals and human beings. Therefore, ethanolic and aqueous extracts of ARs could be attributed to the inhibition of prostaglandin biosynthesis, which in turn inhibits gastrointestinal motility and secretion depending on the composition of saponins, alkaloids, flavonoids, sterols, and terpenes in ARs (96).

### 7.8. Neuroprotective action

Neuronal degeneration plays a vital role in the specific area of the brain in neurodegenerative disorders and the development of the nervous system. Methanolic extract of ARs was tested against kainic acid-induced striatal neuronal and hippocampal damage in mice at 18 mg/kg. After treatment, 10.81–14.0 and 10.0 nmol NADPH oxidized/mg protein in the hippocampus and striatum, respectively, were obtained in glutathione peroxidase (GPx) assays, and 0.83–1.92 and 1.17–2.31 nmol NADPH oxidized/mg protein in the hippocampus and striatum, respectively, were obtained in the glutathione GSH assay. This result suggested that the methanolic extract of ARs plays the role of an antioxidant by attenuating free radical-induced oxidative damage (46). Another study investigated the neuroprotective effects of a methanolic extract from ARs in Swiss albino mice and clinical patients (98). The results demonstrated that cytological effects were significantly higher in the total normal cells of various regions of the hippocampus (CA1, CA3, CA4, and Dg) in the order Dg > CA4 > CA1 > CA3 compared to the control. In a further study, evaluating the neuroprotective effects of ethanolic extracts from ARs significantly reduced ethanol-induced oxidative stress and cognitive impairment in Swiss albino male rats at 100 and 200 mg/kg b.w. (*P* < 0.05, *P* < 0.01), decreased retention transfer latency (RTL), escape latency (EL) and AChE (acetylcholinesterase) activity in the EPM, MWM and TBARS level tests after 21 days. Furthermore, in passive avoidance (PA), novel object recognition (NOR) testing and biochemical studies, such as lipid peroxidation (TBARS) content and AChE tests, ethanolic extract from *A. racemosus* roots markedly increased regency latency (RL), DI (discrimination index) and TSTQ (target quadrant) and time spent in the annuli (TSA) at a dose of 200 mg/kg b.w. (*P* < 0.05, *P* < 0.01) on days 11 and 21 compared to the disease control (99).

### 7.9. Miscellaneous

Asparagus root has been used for over 2,000 years as a medicine prescription (8). AR was used to produce commercial medicinal products in the modern era for clinical settings. Cancer patients taking medications containing AR reported a significant improvement in health outcomes (100). In India, many highly processed asparagus root products and AR powder are popular in the public market (101). In a randomized controlled trial of lactating mothers with symptoms of deficient lactation, Gupta and Shaw found that the oral administration of ARs improved prolactin hormone levels more than threefold compared to the control group (13). No acute toxicity effects were detected. Another study showed improved quality of life for patients with advanced non-small cell lung cancer after treatment with Yiqi Yangyin Jiedu Decoration, a Chinese herbal recipe containing AR (102).

## 8. Potential toxicity of bioactive compounds in AR

Some studies have shown that AR might possess teratogenicity effects (103). For example, methanolic extracts of ARs (100 mg/kg/day for 60 days) showed increased resorption of fetuses, swelling in legs, and intrauterine growth retardation in Charles Foster rats (103). Considering these adverse effects observed in animal studies, AR might harm the human fetus and should be avoided during pregnancy Goel et al. (103).

An acute toxicity study by Kumar et al. (45) reported no fatalities in rats administered 800 mg/kg, 1,600 mg/kg, and 3,200 mg/kg of aqueous extracts of ARs. However, the authors reported significant diuretic activity when 3,200 mg/kg aqueous extracts of ARs were given. The ethanolic and aqueous extracts of AR did not show any toxic effects at doses ranging from 50 mg/kg to 1 g/kg b.w. for 4 weeks in Wistar albino rats (104).

## 9. Conclusion and future prospects

The genus Asparagus is becoming a more valuable plant because of its uses as folk medicine and food. AR is not as popular as asparagus spears because of incomplete research and insufficient evidence regarding their clinical applications. Various studies have concluded that the plant is rich in the alkaloid mimosine, which has anticancer properties. Further efforts are required to elaborate upon the anticarcinogenic effect of mimosine and its isolation at a commercial scale for therapeutic applications in humans. A large number of studies have been performed regarding its antivenom activity. Thus, different asparagus species might have the potential to be used as herbal medicines and functional foods. Furthermore, phytochemical studies have demonstrated the presence of various bioactive compounds as valuable secondary metabolites. The dominant class of bioactive compounds in AR is flavonoids. AR displayed significant pharmacological effects, such as antioxidant, antimicrobial, antiviral, anticancer, anti-inflammatory, and antidiabetic effects, as shown in studies conducted in animals and humans. Some phytochemical compounds have been identified and quantified through NMR, HPL, and TLC. We believe high-throughput techniques, such as Q-TOF-MS, would identify compounds that ordinary instruments might not recognize. This plant could supply a sound basis for developing therapeutic formulations of herbal medicine to treat various health ailments.

## Author contributions

YG: conceptualization, design of the work, writing—review and editing, supervision, and funding acquisition. ZL: visualization, resources, and writing—review and editing. YW, YZ, and HA: conception or design of the work, resources, and investigation. MY: writing—review and editing and supervision. JP, TY, and XZ: data acquisition, formal analysis, conceptualization, and writing—review and editing. AIH and AMA: formal analysis and writing—review and editing. All authors contributed to the article and approved the submitted version.

## Footnotes

1
http://www.webofknowledge.com/

2
https://bibliometric.com
